# Miscellany

**Published:** 1896-03

**Authors:** 


### Miscellany.

### MEDICAL EXPERT TESTIMONY.

THE special committee appointed at the last meeting of the medical society of the State of New York to report upon the most feasible plan by which the present methods of introducing medical expert testimony can be improved, submits the following report:

Your committee recognising the difficulties which lay in the way of formulating any plan within the constitution of the state, have corresponded quite extensively with qualified members of both the legal and medical professions, and believe that in submitting the following preamble and resolution, it presents a conscensus of such opinions held with reference to this subject, which under present constitutional restrictions affords the best method of obtaining medical expert testimony.

WHEREAS, The present method of obtaining medical expert testimony tends to lessen the value of such testimony and to bring the medical profession into disrepute; therefore, be it

*Resolved*, That the medical society of the State of New York would recommend the enactment of a law by the legislature providing for the appointment of experts by the courts, and that only physicians of repute in the particular branch of medical science to which the question calling for expert opinion relates, shall be appointed; that the function of the experts so appointed shall be advisory, and the number thus appointed shall be such as to adequately represent the court, and both sides of the question at issue, as in the judgment of the court shall seem necessary; that the experts so appointed shall have full and free access to all the evidence in the case, as well as access to the plaintiff or defendant in person as the case may be, if the issue involves his mental or physical state. That the expert shall submit to the court for transmission to the jury a report in writing, setting forth their conclusion and the facts in evidence on which such conclusion is based; that the cross-examination of such experts shall be limited to the facts and opinions embraced in their testimony as embodied in their report, and that their compensation shall be fixed by the court at a rate that is reasonable for professional services of such a nature.

Respectfully submitted,

J. B. RANSOM, M. D., E. D. FISHER, M. D.,
CARLOS F. MCDONALD, M. D., S. B. WARD, M. D.,
H. E. ALLISON, M. D., *Committee.*

---

MESSRS. SCHULTZE-BERGE & KOECHL, of New York, announced a change in their firm name. This well-known house will hereafter be styled Victor Koechl & Co. All the business relations, foreign and domestic, of the firm are retained, and the change is more in name than in reality, as Mr. Koechl has been the head of the house for some years.

This house has recently issued a brochure of forty pages on antitoxin, which explains almost every feature of the manufacture and use of serum for preventive inoculation use. This same firm announces also the publication of a new monthly entitled Therapeutic Progress, which will be sent regularly to any physician who sends his name and address to 79 Murray street.

